# Association between serum ferritin and liver stiffness in adults aged ≥20 years: A cross-sectional study based on NHANES

**DOI:** 10.1097/MD.0000000000034838

**Published:** 2023-09-01

**Authors:** Hao Han, Yan Chen, Siqi Zhang, Xiaojuan Ji, Mingli Zhu, Wanyu Ma, Hongfeng Ge, Hailiang Chu

**Affiliations:** a Department of Hematology, Bozhou Hospital Affiliated to Anhui Medical University, Bozhou City, Anhui Province, People’s Republic of China; b Department of General Practice, Wuhu City Second People`s Hospital, Wuhu City, Anhui Province, People’s Republic of China.

**Keywords:** cross-sectional study, iron overload, liver stiffness, NHANES, serum ferritin

## Abstract

The importance of serum ferritin has been demonstrated in many liver diseases, but its relationship with liver stiffness remains unclear. The objective of this study was to investigate the association between serum ferritin levels and participants’ liver stiffness measurement (LSM) in the United States population. We conducted a screening of participants from National Health and Nutrition Examination Survey (NHANES) 2017.1 to 2020.3 to ensure that participants included in this study had complete serum ferritin and LSM information. Association between the independent variable (serum ferritin) and the dependent variable (LSM) was investigated by multiple linear regression and subgroup analysis was performed to identify sensitive individuals, and we subsequently assessed whether there was a non-linear relationship between the 2 using smoothed curve fitting and threshold effect models. The final 7143 participants were included in this study. There was a positive association between participants’ serum ferritin concentration and LSM, with an effect value of (β = 0.0007, 95% confidence interval (CI): 0.0002–0.0011) in the all-adjusted model. The smoothing curve and threshold effect models indicated a non-linear positive correlation between serum ferritin and LSM, which was more pronounced when serum ferritin concentration exceeded 440 ng/mL. Subsequent subgroup analysis showed that this positive correlation was more pronounced in males (β = 0.0007, 95% CI: 0.0001–0.0012), age >60 years (β = 0.00015, 95% CI: 0.0007–0.0023), black participants (β = 0.00018, 95% CI: 0.0009–0.0026), and participants with body mass index (BMI) <25 kg/m^2^ (β = 0.00012, 95% CI: 0.0005–0.0020). In U.S. adults, there was a positive correlation between serum ferritin levels and liver stiffness, which was more pronounced when serum ferritin exceeded 440 ng/mL. Our study suggested that regular serum ferritin testing would be beneficial in monitoring changes in liver stiffness. Male, age >60 years, black participants, and those with a BMI < 25 kg/m^2^ should be of greater consideration.

## 1. Introduction

The normal human liver is soft and elastic, and increased liver stiffness only occurs when the liver is underpinned by chronic substantial injury, a sustained activated inflammatory response and fibrosis formation, with cirrhosis forming at the end stage.^[1]^ There is no single cause of altered liver stiffness, and although the 3 main risk factors of alcohol, obesity and viral hepatitis account for more than 90% of all cases of chronic liver disease,^[2]^ researchers have never stopped exploring the risk factors that alter liver stiffness. It is widely recognized in existing studies that persistent activation of the inflammatory response is an important factor in the eventual progression of chronic liver disease (CLD) to liver fibrosis.^[3]^ Markers that could accurately assess the inflammatory state of the liver have been of interest to researchers, and among the numerous markers, serum ferritin has attracted interest.^[4]^

Ferritin is the major iron storage protein^[5]^ and is also widely acknowledged as an acute phase reactor and marker of acute and chronic inflammation, with elevated serum ferritin often seen in diseases such as chronic kidney disease, rheumatoid arthritis and other autoimmune diseases, acute infections and malignancies.^[6,7]^ Previous studies have shown that ferritin could enter the circulation via the classical endoplasmic reticulum/Golgi-dependent secretory pathway in hepatocytes.^[8,9]^ In addition, another possible mechanism of ferritin secretion involves leakage from damaged cells, which would explain the strong association between serum ferritin and markers of liver cell injury.^[4]^ Therefore, the relevance of serum ferritin to liver disease, as the marker of inflammatory response most closely associated with the liver, has been a matter of interest to researchers.. Higher serum ferritin levels have previously been reported to be associated with a higher prevalence of nonalcoholic fatty liver disease (NAFLD), severe steatosis or advanced fibrosis in patients with NAFLD,^[10]^ and ferritin has also been shown to be an important cofactor in the progression of hepatitis C disease to advanced liver fibrosis or even cirrhosis.^[11,12]^ Although some studies have also reported that serum ferritin levels are less accurate in the diagnosis of liver fibrosis due to NAFLD,^[13]^ the potential association between serum ferritin and liver disease is unquestionable. As mentioned earlier, changes in liver stiffness are not caused by a single disease or factor. Although previous studies have provided liver stiffness measurements (LSM) for the diagnosis of liver fibrosis,^[14–16]^ they remain controversial. Therefore, studying the correlation between serum ferritin and a particular disease alone would be very limited. In addition, studies on serum ferritin and liver stiffness, especially large cross-sectional studies, is still scarce. Whether the correlation between serum ferritin and liver stiffness in different conditions has been specifically confirmed in cross-sectional studies is still unknown.

NHANES database is a rigorous, scientific database that includes samples that are representative for the United States population and has an adequate sample size and is often used as an important tool in cross-sectional studies.^[17]^ The aim of this study was to explore the association between serum ferritin and liver stiffness using information from the NHANES database. In this study, we did not exclude any particular disease that might affect liver stiffness, but instead adjusted as a covariate in the regression model to verify an independent positive association between serum ferritin and liver stiffness, and analyzed this positive association in a population with different characteristics.

## 2. Methods

### 2.1. Data source

NHANES, originally known as the National Health and Nutrition Examination Survey, was first started in the 1960 with a complex sampling design of stratified, multi-stage, and block group participants from the general United States population to ensure a nationally representative sample of participants. The NHANES aims to obtain information on participants’ demographics, diet, body examination and laboratory tests through questionnaires, body examinations and laboratory tests to assess the nutritional and medical conditions of the United States citizen population. NHANES updates data at a frequency of a 2-year time cycle. NHANES began including data on participants’ liver ultrasound transient elastography in 2017, but the NHANES program suspended field operations in March 2020 due to the Coronavirus disease 2019 pandemic, so our study relied on NHANES 2017.1 to 2020.3 data. NHANES was conducted by the National Center for Health Statistics of the Centers for Disease Control and Prevention, and the National Center for Health Statistics Research Ethics Review Committee endorsed the NHANES survey protocol, so ethical review of this study was exempt. The NAHENS project provided written informed consent for all participants included in the survey. The study followed relevant guidelines and regulations.

### 2.2. Participants

**Figure 1.**
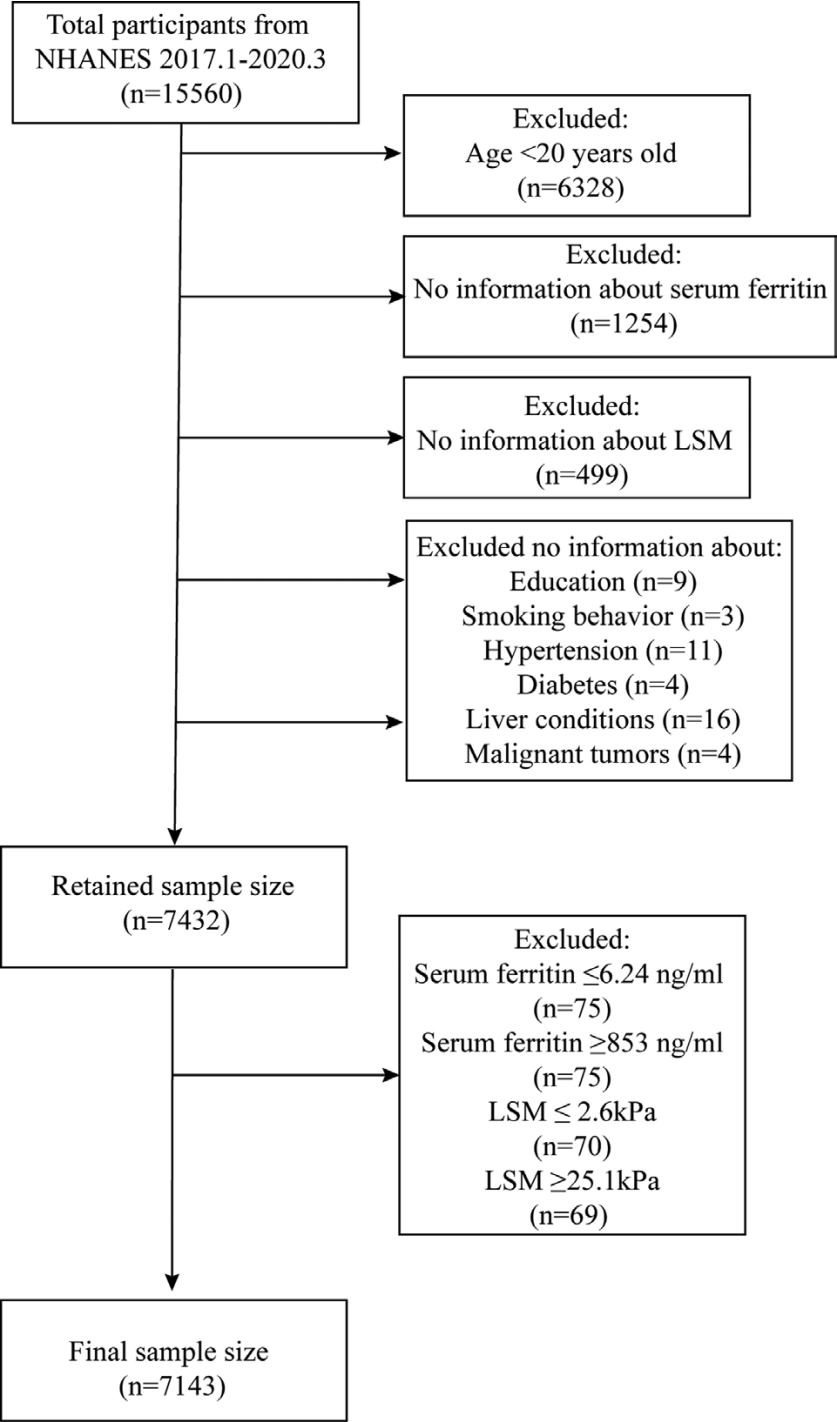
Flow chart for participants.

A total of 15,560 participants enrolled in the NHANES 2017.1 to 2020.3 survey. We first excluded participants under the age of 20 years (n = 6328), retaining 7432 participants with complete serum ferritin and LSM information. Before determining the final study sample size, we performed a smoothed curve fit for serum ferritin and LSM on the 7432 participants and identified the presence of outliers (Supplementary Fig. 1, http://links.lww.com/MD/J535). We first ranked serum ferritin and LSM in ascending order from smallest to largest and performed subsequent processing. Serum ferritin concentrations of 6.24 and 853 ng/mL were first determined for the ranked 1% and 99%, respectively, and participants with serum ferritin ≤6.24 ng/mL (n = 75), and ≥853 ng/mL (n = 75) were removed. The ranking of 1% and 99% LSM 2.6 and 25.1 kPa were also identified, and participants with LSM ≤ 2.6 kPa (n = 70), and over ≥ 25.1 kPa (n = 69) were removed. A total of 7143 participants were eventually included in this study (Fig. 1).

### 2.3. Dependent and independent variables

Serum ferritin was obtained as an independent variable from laboratory examination data. The method for the measurement of serum ferritin on the Roche Cobas e601 was a sandwich principle with a total duration time of 18 minutes. The 1st incubation used 10 μL of sample, a ferritin-specific antibody and a labeled ferritin-specific antibody to form a sandwich complex. The 2nd incubation occurred after the addition of microparticles that caused the complex to bind to the solid phase. The reaction mixture was aspirated into the measuring cell where the microparticles were magnetically captured onto the surface of the electrode. Unbound substances were then removed. Application of a voltage to the electrode then induced chemiluminescent emission which was measured by a photomultiplier. Results were determined via a calibration curve. A detailed description of the laboratory methods used can be found in the laboratory methods documentation section (https://wwwn.cdc.gov/Nchs/Nhanes/2017-2018/P_FERTIN.htm).

The LSM as a strain was derived from body examination data. Ultrasound transient elastography measurements were performed by trained medical technicians at the NHANES Mobile Examination Centre with a FibroScan type V2 touch equipped with a medium or extra-large probe. Participants who could not lie on the examination table, were pregnant at the time of the examination, had an electronic medical device implanted, wore a bandage or had damage to the measurement site were excluded from the examination. A detailed description of the laboratory methods used can be found at the NHANES website (https://wwwn.cdc.gov/nchs/nhanes/search/datapage.aspx?Component=Examination&CycleBeginYear=2017).

### 2.4. Covariates

Theoretically, factors with potential effects on serum ferritin levels and LSM should be adjusted for as covariates in a multiple linear regression model, but such study conditions may not be met in practice. Therefore, based on previous studies, we have used well known variables as covariates in this study.^[18–20]^

Demographic information included age, gender, race, ratio of family income to poverty, education level and body mass index (BMI) was obtained from the body examination and was calculated as BMI = weight (kg)/height (m)^2^. Smoking and alcohol consumption were obtained from the questionnaire. Smoking conditions were categorized as current, ever and never, and alcohol consumption was divided into 2 subgroups (≤2 and ≥3 drinks per day), based on the average number of drinks per day in the previous 12 months. Medical conditions were obtained from questionnaires, and hypertension, diabetes, malignancy, liver conditions (viral hepatitis, fatty liver, cirrhosis, autoimmune liver disease, other liver diseases), and history of receiving blood transfusions were used as covariates in the model and adjusted to exclude the effect of disease on the independent and dependent variables. Participants’ daily intake of total nutrients (iron and energy) was obtained from a questionnaire and calculated as the mean of the sum of the nutrient values answered on the first and second day. Sedentary minutes were obtained from the physical activity questionnaire and we collected directly the time participants were sedentary and inactive (min). Alanine aminotransferase (U/L), aspartate aminotransferase (U/L), and gamma glutamyl transferase (IU/L) were obtained with standard biochemical data from laboratory tests and all participants were asked to fast for 9 hours and evaluated by staff for fasting status before blood samples were drawn. NHANES only performed analysis on samples that met the conditions of the laboratory tests. Both serum ferritin and liver stiffness have been associated with chronic inflammation,^[21]^ so we obtained data on high-sensitivity C-reactive protein from the laboratory data. Blood lead and blood cadmium levels have been clearly associated with liver fibrosis,^[22–24]^ so we also collected data on these 2 heavy metals. All participants in this study provided adequate serum samples, which were processed, stored and transported to the Advanced Research Diagnostic Laboratory at the University of Minnesota in Minneapolis, Minnesota for analysis, and the data obtained from the analyses were confirmed. All covariates are listed in Table 1.

**Table 1:** Characteristics of the participants.

Characteristics	Cohort A	Cohort B	*P* value
Sample size	4523	2620	
Gender, (%)			<.001
Male	36.04	72.33	
Female	63.96	27.67	
Age (yr)	49.30 ± 17.84	53.28 ± 16.08	<.001
Stratified by age (yr), (%)			<.001
<40	34.58	22.98	
40–60	32.24	36.15	
>60	33.19	40.88	
Race			.044
White	36.13	33.40	
Black	24.76	26.72	
Other race	24.76	26.72	
Education			.191
Less than high school	17.75	19.05	
High school	23.86	24.69	
More than high school	58.39	56.26	
PIR			.086
<1.35	25.51	22.86	
1.35–3.45	32.39	33.02	
>3.45	28.79	30.34	
Not recorded	13.31	13.78	
BMI (kg/m^2^)	29.75 ± 7.52	30.11 ± 6.67	<.001
Stratified by BMI (kg/m^2^), (%)			<.001
<25	27.19	21.64	
25–30	31.95	34.27	
>30	40.86	44.08	
Smoking behavior			<.001
Current	17.40	19.58	
Ever	22.15	26.83	
Never	60.45	53.59	
Alcohol consumption			<.001
≤2 drinks/d	46.89	41.45	
>2 drinks/d	19.66	28.44	
Not recorded	33.45	30.11	
Sedentary minutes (min)	327.54 ± 201.18	333.03 ± 201.14	.235
Stratified by Sedentary minutes (min), (%)			.282
≤300	46.58	45.27	
>300	53.42	54.73	
Hypertension, n (%)			<.001
Yes	35.66	41.91	
No	64.34	58.09	
Diabetes, n (%)			.003
Yes	13.82	16.22	
No	83.46	80.34	
Borderline	2.72	3.44	
Liver conditions			<.001
Yes	4.47	6.22	
No	95.53	93.78	
Malignant tumors			.212
Yes	10.35	9.43	
No	89.65	90.57	
Received blood transfusion			.002
Yes	11.61	9.27	
No	87.31	89.12	
Not recorded	1.08	1.60	
Energy (Kcal)			<.001
≤3000	23.37	18.28	
>3000	55.85	61.34	
Not recorded	20.78	20.38	
Iron (mg)			<.001
≤20	29.16	24.92	
>20	50.06	54.69	
Not recorded	20.78	534	
ALT (U/L)	18.91 ± 10.90	27.52 ± 20.91	<.001
AST (U/L)	20.02 ± 8.79	24.22 ± 14.06	<.001
GGT (IU/L)	26.04 ± 34.71	41.66 ± 69.93	<.001
hs-CRP (mg/L)	3.80 ± 6.35	4.35 ± 10.74	.006
Blood lead (µg/dL)	1.12 ± 1.09	1.35 ± 1.54	<.001
Blood cadmium (µg/L)	0.48 ± 0.56	0.45 ± 0.53	<.001
LSM (kPa)	5.43 ± 2.50	5.98 ± 2.84	<.001

Mean ± SD for continuous variables: *P* value was calculated by weighted linear regression model. % for categorical variables: *P* value as calculated by weighted Chi-square test.

BMI = body mass index, hs-CRP = high-sensitivity C-reactive protein, LSM = liver stiffness measurement, PIR = ratio of family income to poverty.

### 2.5. Statistical analysis

The NHANES database provides data in XPT format and all data extraction and analysis was performed in R (http://www.R-project.org) and EmpowerStats (http://www.empowerstats.com). Continuous variables were displayed as mean ± standard deviation and categorical variables were displayed as percentages (%). The presence of missing values for covariates in cross-sectional studies is inevitable, and in order to retain more information on independent and respondent variables, missing values for covariates were processed. If the missing value of a continuous variable was within 10% of the total sample, we used the mean as a substitute, otherwise we grouped the continuous variable according to certain rules and set the missing values in a separate group. Missing values for categorical variables were directly removed if they did not exceed a sample size of 20, otherwise we grouped them separately. Multiple linear regression was used to explore the association between serum ferritin and LSM, and 3 models were generated based on the covariates adjusted for in the model (model 1: non-adjusted model; model 2: age, gender, race were adjusted; model 3: all covariates in Table 1 were adjusted). Continuous variables were displayed as mean ± standard deviation and categorical variables were displayed as percentages (%). The presence of missing values for covariates in cross-sectional studies is inevitable, and in order to retain more information on independent and respondent variables, missing values for covariates were processed. If the missing value of a continuous variable was within 10% of the total sample, we used the mean as a substitute, otherwise we grouped the continuous variable according to certain rules and set the missing values in a separate group. Missing values for categorical variables were directly removed if they did not exceed a sample size of 20, otherwise we grouped them separately. Multiple linear regression was used to explore the association between serum ferritin and LSM, and 3 models were generated based on the covariates adjusted for in the model (model 1: non-adjusted model; model 2: age, gender, race were adjusted; model 3: all covariates in Table 1 were adjusted). We conducted subgroup analysis based on participants’ gender (male, female), age (years) (<40, 40–60, >60), race (white, black, other races), and BMI (kg/m^2^) (<25, 25–30, >30) to identify cohort characteristics that warranted our attention. We then verified whether there was a non-linear relationship between serum ferritin and LSM with smoothed curve fitting and threshold effect models. A non-linear relationship was suggested when the log-likelihood ratio (<0.05) was present.

## 3. Results

### 3.1. Characteristics of participants

As both the independent and response variables were continuous, we divided the participants into 2 cohorts (cohort A: ≤150.215; cohort B: >150.215) using the mean value of serum ferritin concentration (150.215 ng/mL) as the node. A total of 7143 participants were included in this study, 4523 in cohort A and 2620 in cohort B. Cohort B had a higher LSM than cohort A (*P* < .05) (Table 1).

### 3.2. The association between serum ferritin and LSM

As mentioned previously, outliers were removed to exclude the effect of outliers on the association between serum ferritin and LSM, but we equally analyzed the samples without excluded outliers and the results suggested a positive association between serum ferritin and LSM, with an effect value of (β = 0.0013, 95% CI [confidence interval]: 0.0006–0.0019) in the all-adjusted model (Supplementary Table 1, http://links.lww.com/MD/J536). A positive association between serum ferritin and LSM was found in all models generated in the final sample, with an effect value of (β = 0.0007, 95% CI: 0.0002–0.0011) in the all-adjusted model (Table 2). The above results indicated that the presence of outliers, while not altering the positive correlation between serum ferritin and LSM, potentially exaggerated the effect values between the 2, so we considered that samples with outliers removed might be more appropriate for assessing the association between serum ferritin and LSM. Subsequent subgroup analysis showed that the statistically significant cohorts were male (β = 0.0007, 95% CI: 0.0001–0.0012), >60 years old (β = 0.00015, 95% CI: 0.0007–0.0023), black (β = 0.00018, 95% CI: 0.0009–0.0026), BMI < 25 kg/m^2^ (β = 0.00012, 95% CI: 0.0005–0.0020), 25 kg/m^2^ < BMI < 30 kg/m^2^ (β = 0.0007, 95% CI: 0.0001–0.0013) (Table 2).

**Table 2:** The association between serum ferritin (ng/mL) and LSM (kPa) and the results of subgroup analysis.

	Model 1, β (95% CI)	Model 2, β (95% CI)	Model 3, β (95% CI)
Serum ferritin (ng/mL)	0.0025 (0.0021, 0.0030)	0.0018 (0.0013, 0.0023)	0.0007 (0.0002, 0.0011)
Subgroups			
Gender			
Male	0.0017 (0.0011, 0.0023)	0.0017 (0.0011, 0.0023)	0.0007 (0.0001, 0.0012)
Female	0.0029 (0.0021, 0.0037)	0.0019 (0.0010, 0.0027)	0.0007 (−0.0001, 0.0015)
Age (yr)			
<40	0.0026 (0.0019, 0.0034)	0.0017 (0.0008, 0.0026)	−0.0003 (−0.0011, 0.0005)
40–60	0.0020 (0.0012, 0.0027)	0.0017 (0.0009, 0.0026)	0.0002 (−0.0005, 0.0010)
>60	0.0021 (0.0013, 0.0029)	0.0018 (0.0010, 0.0027)	0.0015 (0.0007, 0.0023)
Race			
White	0.0023 (0.0014, 0.0031)	0.0017 (0.0008, 0.0026)	0.0002 (−0.0007, 0.0010)
Black	0.0034 (0.0026, 0.0042)	0.0028 (0.0019, 0.0038)	0.0018 (0.0009, 0.0026)
Other race	0.0021 (0.0014, 0.0027)	0.0011 (0.0004, 0.0018)	0.0002 (−0.0005, 0.0009)
BMI (kg/m^2^)			
<25	0.0028 (0.0021, 0.0034)	0.0019 (0.0012, 0.0026)	0.0012 (0.0005, 0.0020)
25–30	0.0022 (0.0016, 0.0028)	0.0016 (0.0009, 0.0022)	0.0007 (0.0001, 0.0013)
>30	0.0023 (0.0015, 0.0031)	0.0013 (0.0004, 0.0022)	0.0003 (−0.0006, 0.0012)

Model 1: No covariates were adjusted. Model 2: Age, gender and race were adjusted. Model 3: All the covariates in Table 1 were adjusted.

BMI = body mass index, LSM = liver stiffness measurement.

*In the subgroup analysis stratified by each covariate, the model is not adjusted for the stratification variable itself.

### 3.3. Smoothing curve fitting and threshold effect analysis

**Figure 2.**
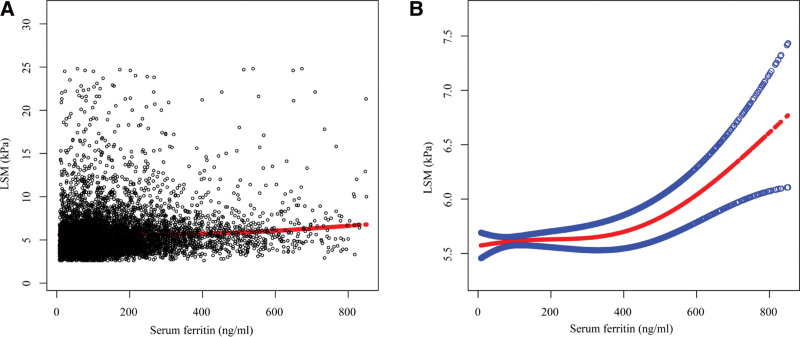
The association between serum ferritin (ng/mL) and LSM (kPa). (A) Each black point represents a sample. (B) Solid rad line represents the smooth curve fit between variables. Blue bands represent the 95% of confidence interval from the fit. *All the covariates in Table 1 were adjusted. LSM = liver stiffness measurement.

**Figure 3.**
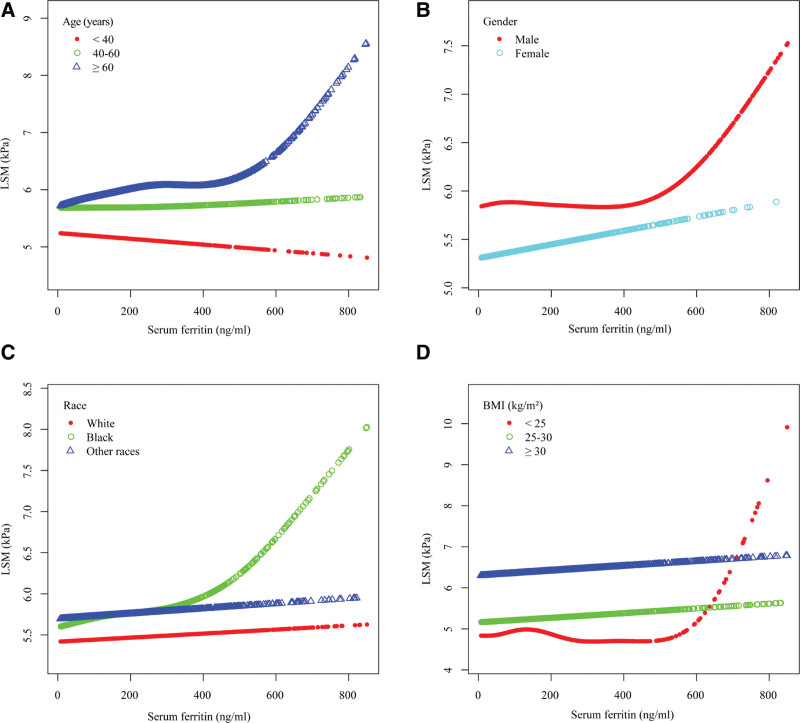
The association between serum ferritin (ng/mL) and LSM (kPa), stratified by age (yr), gender, race and BMI (kg/m^2^). *All the covariates in Table 1 were adjusted. *In the subgroup analysis stratified by each covariate, the model is not adjusted for the stratification variable itself. LSM = liver stiffness measurement.

We verified that this positive correlation between serum ferritin and LSM was non-linear with a smooth curve fitting model (Fig. 2) and that the positive correlation between serum ferritin and LSM was more pronounced when serum ferritin exceeded the inflection point of 440 ng/mL (β = 0.0029, 95% CI: 0.0013–0.0046) with a threshold effect model (Supplementary Table 2, http://links.lww.com/MD/J537). We also performed smoothed curve fitting and threshold effect analysis for each subgroup in the subgroup analysis, and the non-linear positive association between serum ferritin and LSM was also present in the subgroups of male, >60 years old, and BMI < 25 kg/m^2^, with inflection points of 525, 486, 452, and 389 ng/mL for serum ferritin, respectively (Tables 3–6 and Fig. 3).

## 4. Discussion

Previous studies have focused mainly on the correlation between serum ferritin and liver fibrosis,^[25,26]^ but the development of liver fibrosis is a subtle process and early control of risk factors for elevated liver stiffness is necessary to benefit a larger population. The factors influencing the development of altered liver stiffness are diverse, which means that exploration and identification of potential risk factors is essential. Increases in liver stiffness also affect changes in serum ferritin concentrations,^[5]^ and it can be argued that the 2 affect each other. However, in previous studies both serum ferritin concentrations and liver stiffness were at high values (cirrhotic or fibrotic stage), and the conclusions obtained in this case were often somewhat limited. In the present study, we demonstrated a positive correlation between serum ferritin concentration and liver stiffness, and we also found that participants may be more susceptible to higher liver stiffness when serum ferritin concentrations exceed 440 ng/mL, a finding that would be similar to previous studies^[18,27,28]^ and would again be a good addition. Furthermore, it was interesting that in our subgroup analysis results, the association between serum ferritin concentration and liver stiffness was only statistically significant in specific subgroups (male, age >60 years, black, BMI < 25 kg/m^2^, 25 kg/m^2^ < BMI < 30 kg/m^2^).

The difference in serum ferritin concentrations by gender and age could be explained by a number of possible reasons. Firstly, based on the results in Table 1, we found that participants who were male and aged >60 years had higher serum ferritin concentrations, which was similar to the results of previous studies.^[29,30]^ Serum ferritin concentrations are regulated by a number of factors, of which the influence of sex hormones is not negligible. In the case of testosterone, it is known to be one of the most active androgens and plays an important role in erythropoiesis and iron metabolism.^[31]^ Testosterone is inhibited by hepcidin, a hormone synthesized by the liver that regulates iron absorption and mobilization, leading to increased serum free iron and increased serum ferritin concentrations.^[32,33]^ In addition to hormonal factors, serum ferritin may be associated with the prevalence of insulin resistance (IR), chronic inflammatory diseases and tumors in older age groups.^[34]^

Previous studies have shown that the black race is a special presence in racially diverse United States population, after excluding the effects of gender and age.^[35]^ Black racial U.S. population tends to have higher serum ferritin levels, which is associated with them having a higher prevalence of metabolic disorders.^[36]^ In addition to this, this racial specificity of high serum ferritin concentrations is associated with the amount of iron in the black racial diet and human leukocyte antigen-linked genes that differ from other races, where human leukocyte antigen-linked genes are known to be important in causing iron overload.^[37]^

As mentioned previously, hepcidin is essential for the metabolism of serum ferritin. However, obesity-associated inflammation triggers abnormal ferritin synthesis, leading to disruption of iron homeostasis and the development of iron overload.^[38]^ It follows that obese people may have higher serum ferritin levels, which is similar to the results of our study (Table 1 and Fig. 3). In this study, we found several interesting results. First, in the subgroup analysis, the positive association between serum ferritin and liver stiffness was more pronounced in people with lower BMI, especially those with BMI < 25 kg/m^2^. Second, in the threshold effects model, the inflection point for serum ferritin was lower in those with BMI < 25 kg/m^2^ compared to other subgroups with statistically significant differences. We might therefore conjecture that although serum ferritin concentrations would not be higher in people with low BMI, the effect on their liver stiffness would be more pronounced. There are still no definitive studies to explain such a result, but previous studies have shown that people with NAFLD with low BMI have a greater tendency to develop visceral fat accumulation,^[39]^ which produces inflammatory cytokines such as interleukin-6 (IL-6) and tumor necrosis factor, leading to more severe IR.^[40]^ The effects of IR, serum ferritin and liver stiffness are complex and reciprocal.^[41,42]^ In any case, we believe that the potential mechanism for a more significant effect on liver stiffness in low BMI populations with iron overload still needs to be confirmed by more follow-up studies.

Overall, serum ferritin has been receiving attention as the biomarker most closely related to the liver. The strength of our study was to establish a cross-sectional study with a large sample and demonstrated the close association of serum ferritin with liver stiffness. With the development of deep learning techniques in imaging disciplines, progress has been made in the noninvasive diagnosis of many diseases, such as heart failure and tumors.^[43–45]^ We believe that there is a trend towards fitting liver stiffness using validated variables such as serum ferritin. However, there were still some limitations to this study. First, the presence of missing values for covariates is a problem that cannot be avoided in cross-sectional studies, and although we have treated missing values for covariates according to certain rules, we could not guarantee that the association between the independent and the dependent variables is completely independently of each other, just as the most perfect cross-sectional studies still cannot explain causality.^[46]^ Second, in order to better illustrate the correlation between the independent and the dependent variables, we have excluded the effect of outliers in our study based on a smoothed curve model, which may be justified in practice by the presence of what we call “outliers,” which means that the independent and the dependent variables in this study did not completely cover the distribution of the whole data. This troubling aspect is then unavoidable for researchers conducting cross-sectional studies.^[47]^ Third, the potential factors that might have an effect on serum ferritin and liver stiffness are multifaceted, and although we included relevant covariates in adjusted models based on previous studies, there is no guarantee of bias from other potential covariates. Finally, this is a study based on a cohort of U.S. adults, and the applicability of the current results to other age groups and national populations requires follow-up studies. In any case, due to these limitations, the findings of this study are to be treated with caution.

## 5. Conclusions

A positive association was found between serum ferritin and liver stiffness in the United States population and this positive association was more pronounced among males, age >60 years, black participants, and those with a BMI < 25 kg/m^2^. However, the positive correlation was non-linear and it was alarming that the increase in liver stiffness was higher when serum ferritin exceeded 440 ng/mL, while the inflection points for serum ferritin were 525, 486, 452, and 389 ng/mL in males, >60 years and BMI < 25 kg/m^2^ respectively. Our study demonstrated the value of serum ferritin for liver stiffness and provides a reference for more in-depth subsequent studies.

**Table 3:** Threshold effect analysis of serum ferritin (ng/mL) and LSM (kPa) stratified by gender.

Gender	Male	Female
Model 1, β (95% CI)		
Linear effect model	0.0007 (0.0001, 0.0012)	0.0007 (−0.0001, 0.0015)
Model 2, β (95% CI)		
Inflection point (K)	525	29.5
<K	−0.0001 (−0.0008, 0.0006)	−0.0076 (−0.0216, 0.0064)
>K	0.0061 (0.0032, 0.0090)	0.0008 (−0.0000, 0.0017)
LLR	<0.001	0.24

Model 1: Linear effects model; Model 2: Non-linear effects model.

LLR = log-likelihood ratio, LSM = liver stiffness measurement.

*All the covariates in Table 1 were adjusted.

*In the subgroup analysis stratified by each covariate, the model is not adjusted for the stratification variable itself.

**Table 4:** Threshold effect analysis of serum ferritin (ng/mL) and LSM (kPa) stratified by age (yr).

Age (yr)	<40	40–60	>60
Model 1, β (95% CI)			
Linear effect model	−0.0003 (−0.0011, 0.0005)	0.0002 (−0.0005, 0.0010)	0.0015 (0.0007, 0.0023)
Model 2, β (95% CI)			
Inflection point (K)	79.7	267	486
<K	0.0024 (−0.0015, 0.0064)	−0.0005 (−0.0019, 0.0009)	0.0006 (−0.0004, 0.0016)
>K	−0.0006 (−0.0015, 0.0003)	0.0010 (−0.0004, 0.0025)	0.0061 (0.0026, 0.0096)
LLR	0.164	0.194	0.008

Model 1: Linear effects model; Model 2: Non-linear effects model.

LLR = log-likelihood ratio, LSM = liver stiffness measurement.

*All the covariates in Table 1 were adjusted.

*In the subgroup analysis stratified by each covariate, the model is not adjusted for the stratification variable itself.

**Table 5:** Threshold effect analysis of serum ferritin (ng/mL) and LSM (kPa) stratified by race.

Race	White	Black	Other race
Model 1, β (95% CI)			
Linear effect model	0.0002 (−0.0007, 0.0010)	0.0018 (0.0009, 0.0026)	0.0002 (−0.0005, 0.0009)
Model 2, β (95% CI)			
Inflection point (K)	71.2	452	365
<K	0.0030 (−0.0031, 0.0092)	0.0007 (−0.0004, 0.0019)	−0.0004 (−0.0014, 0.0006)
>K	−0.0000 (−0.0010, 0.0009)	0.0060 (0.0028, 0.0092)	0.0014 (−0.0004, 0.0032)
LLR	0.354	0.006	0.145

Model 1: Linear effects model; Model 2: Non-linear effects model.

LLR = log-likelihood ratio, LSM = liver stiffness measurement.

*All the covariates in Table 1 were adjusted.

*In the subgroup analysis stratified by each covariate, the model is not adjusted for the stratification variable itself.

**Table 6:** Threshold effect analysis of serum ferritin (ng/mL) and LSM (kPa) stratified by BMI (kg/m^2^).

BMI (kg/m^2^)	<25	25–30	>30
Model 1, β (95% CI)			
Linear effect model	0.0012 (0.0005, 0.0020)	0.0007 (0.0001, 0.0013)	0.0003 (−0.0006, 0.0012)
Model 2, β (95% CI)			
Inflection point (K)	389	275	28.9
<K	−0.0004 (−0.0013, 0.0006)	−0.0001 (−0.0012, 0.0011)	−0.0129 (−0.0397, 0.0139)
>K	0.0062 (0.0040, 0.0084)	0.0015 (0.0003, 0.0028)	0.0004 (−0.0005, 0.0013)
LLR	<0.001	0.11	0.32

Model 1: Linear effects model; Model 2: Non-linear effects model.

LLR = log-likelihood ratio, LSM = liver stiffness measurement.

*All the covariates in Table 1 were adjusted.

*In the subgroup analysis stratified by each covariate, the model is not adjusted for the stratification variable itself.

## Acknowledgments

The authors thank the staff and the participants of the NHANES study for their valuable contributions.

## Author contributions

**Conceptualization:** Hao Han, Hongfeng Ge, Hailiang Chu.

**Data curation:** Hao Han, Siqi Zhang, Xiaojuan Ji, Wanyu Ma, Hailiang Chu.

**Formal analysis:** Hao Han, Hongfeng Ge, Hailiang Chu.

**Investigation:** Hao Han.

**Methodology:** Yan Chen, Wanyu Ma, Hailiang Chu.

**Project administration:** Hongfeng Ge, Hailiang Chu.

**Software:** Mingli Zhu.

**Supervision:** Siqi Zhang, Xiaojuan Ji.

**Validation:** Yan Chen, Xiaojuan Ji.

**Visualization:** Xiaojuan Ji.

**Writing – original draft:** Hao Han.

**Writing – review & editing:** Mingli Zhu, Hongfeng Ge, Hailiang Chu.

## Supplementary Material
