# Supplementary material for: Association between serum ferritin and liver stiffness in adults aged ≥20 years: A cross-sectional study based on NHANES
Source: Medicine (Baltimore). 2023 Sep 1;102(35):e34838. doi: 10.1097/MD.0000000000034838 (PMC10476712; doi:10.1097/MD.0000000000034838)

Supplementary figure 1. Association between serum ferritin (ng/ml) and LSM (kPa) containing outliers.(a) Each black point represents a sample. (b) Solid rad line represents the smooth curve fit between variables. Blue bands represent the 95% of confidence interval from the fit. *All the covariates in Table 1 were adjusted.

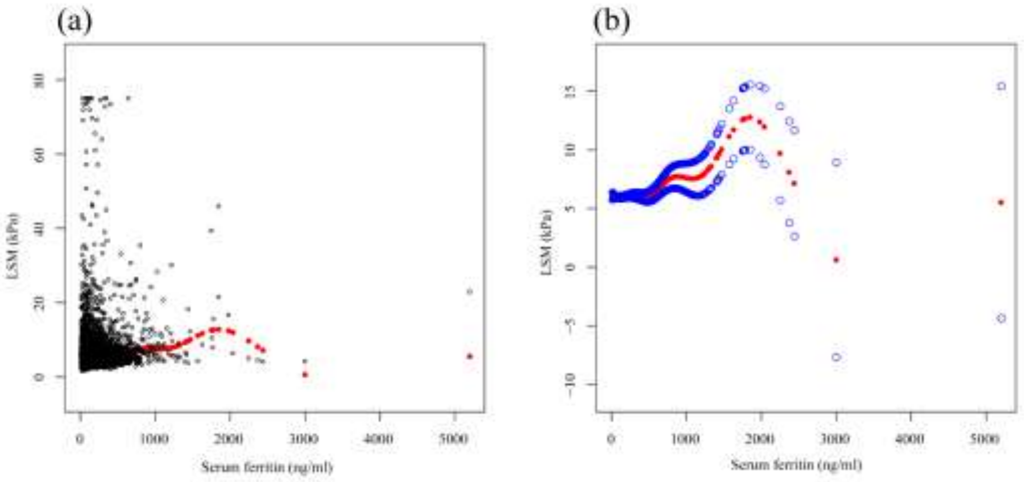

Supplement: Supplementary file 1 [file medi-102-e34838-s001.pdf]
