# Supplementary material for: Association between serum ferritin and liver stiffness in adults aged ≥20 years: A cross-sectional study based on NHANES
Source: Medicine (Baltimore). 2023 Sep 1;102(35):e34838. doi: 10.1097/MD.0000000000034838 (PMC10476712; doi:10.1097/MD.0000000000034838)
Supplement: Supplementary file 2 [file medi-102-e34838-s002.pdf]

**Supplementary Table 1.** Association between serum ferritin (ng/ml) and LSM (kPa) containing outliers

| | Model 1, β (95% CI) | Model 2, β (95% CI) | Model 3, β (95% CI) |
|---|---|---|---|
| Serum ferritin (ng/ml) | 0.0034 (0.0028, 0.0040) | 0.0027 (0.0020, 0.0034) | 0.0013 (0.0006, 0.0019) |

Model 1: No covariates were adjusted. Model 2: Age, gender, race were adjusted. Model 3: All the covariates in Table 1 were adjusted.
