# Supplementary material for: Association between serum ferritin and liver stiffness in adults aged ≥20 years: A cross-sectional study based on NHANES
Source: Medicine (Baltimore). 2023 Sep 1;102(35):e34838. doi: 10.1097/MD.0000000000034838 (PMC10476712; doi:10.1097/MD.0000000000034838)
Supplement: Supplementary file 3 [file medi-102-e34838-s003.pdf]

Supplementary Table 2. Threshold effect analysis of serum ferritin (ng/ml) and LSM (kPa)

Outcome:	LSM (kPa)	*P*-value
Model 1, β (95% CI)		
Linear effect model	0.0007 (0.0002, 0.0011)	0.004
Model 2, β (95% CI)		
Inflection point (K)	440	
< K	0.0001 (-0.0005, 0.0007)	0.71
> K	0.0029 (0.0013, 0.0046)	< 0.001
LLR	0.006	

Model 1: Linear effects model; Model 2: Non-linear effects model.

*All the covariates in Table 1 were adjusted.
